# Forensic age estimation by MRI of the knee – comparison of two classifications for ossification stages in a German population

**DOI:** 10.1007/s00414-024-03281-5

**Published:** 2024-07-04

**Authors:** V Malokaj, MF Wernsing, SN Kunz, M Beer, D Vogele

**Affiliations:** 1 Department of Diagnostic and Interventional Radiology, University Hospital Ulm, Albert-Einstein-Allee 23, 89081 Ulm, Germany; 2 Institute of Forensic Medicine, Ulm University, Ulm, Germany

**Keywords:** Forensic age estimation, Knee MRI, Ossification stages, Bone age

## Abstract

**Aim and objectives:**

In forensic age estimation e.g. for judicial proceedings surpassed age thresholds can be legally relevant. To examine age related differences in skeletal development the recommendations by the Study Group on Forensic Age Diagnostics (AGFAD) are based on ionizing radiation (among others orthopantomograms, plain x-rays of the hand). Vieth et al. and Ottow et al. proposed MRI-classifications for the epiphyseal-diaphyseal fusion of the knee joint to define different age groups in healthy volunteers. The aim of the present study was to directly compare these two classifications in a large German patient population.

**Materials and methods:**

MRI of the knee joint of 900 patients (405 female, 495 male) from 10 to 28 years of age were retrospectively analyzed. Acquired T1-weighted turbo spin-echo sequence (TSE) and T2-weighted sequence with fat suppression by turbo inversion recovery magnitude (TIRM) were analyzed for the two classifications. The different bony fusion stages of the two classifications were determined and the corresponding chronological ages assigned. Differences between the sexes were analyzed. Intra- and inter-observer agreements were determined using Cohen’s kappa.

**Results:**

With the classification of Ottow et al. it was possible to determine completion of the 18th and 21st year of life in both sexes. With the classification of Vieth et al. completion of the 18th year of life for female patients and the 14th and 21st year of life in both sexes could be determined. The intra- and inter-observer agreement levels were very good (κ > 0.82).

**Conclusion:**

In the large German patient cohort of this study it was possible to determine the 18th year of life with for both sexes with the classification of Ottow et al. and for female patients with the classification of Vieth et al. It was also possible to determine the 21st year of life for all bones with the classification of Ottow et al. and for the distal femur with the classification of Vieth et al.

## Introduction

According to the World Migration Report 2020, the proportion of refugees has increased by 77% since the 1990s. A great number of asylum seekers are unable to present valid proof of identity that could be used to determine the age of the respective persons [1, 2]. Without valid identification documents, it is difficult to determine the correct age of young refugees. The correct age is not only crucial for their future, as it determines access to education, protection measures and assistance, but also of central importance in legal issues. For many legal decisions, exceeding or falling below legally defined age limits is crucial, for example the completion of the 18th year of life [3]. In the absence of valid identification documents, authorities and courts can request forensic age estimation. An increase in the number of reports for forensic age diagnostics has been observed in recent years in correlation with increasing migration [4].

While the human skeleton undergoes developmental processes, the determination of an individual’s bone age can be achieved through the assessment of epiphyseal-diaphyseal fusion [5]. The epiphysis, which is primarily composed of hyaline cartilage during the growth phase, can be visualized through various imaging modalities. This visualization allows for the alignment of bone age with chronological age. To facilitate this process, recommendations by the Study Group on Forensic Age Diagnostics of the German Society of Legal Medicine (AGFAD) consist of a physical examination, an X-ray examination of the left hand, and a dental examination. Additionally, if necessary, the clavicles can additionally be examined in more detail using computed tomography (CT) [6, 7].

In many legal systems the utilization of ionizing radiation in healthy persons is controversial. Not only due to the heightened sensitivity of pediatric tissue to radiation, the ionizing radiation must also be used on a restrictive basis [8, 9]. Ultrasound and MRI have been examined as possible alternatives without the use of ionizing radiation [10–14]. Despite MRI of the hand, MRI of the knee was also proposed as a possible tool for forensic age estimation [1, 5, 15]. Existing studies are based on MRI examinations of healthy volunteers. To our best knowledge, our study is the first to compare two classification systems for ossification stages of the knee joint in a large German patient population.

Based on the findings above, we hypothesized that:

1. MRI of the knee can be used as radiation free alternative for forensic age estimation.
2. Classifications developed on healthy volunteers can be applied to a large patient cohort.
3. MRI application can be included in current assessment practice.

## Materials and methods

### Study population

In this retrospective study, 405 MRI examinations of female patients and 495 MRI examinations of male patients were evaluated, whose age at the time of examination ranged from 10 years to 28 years. The indications and questions for MRI examinations were divided into a total of 14 categories and are shown in Table 1. Those 900 MRI examinations were performed at a tertiary hospital over a period of 8 years (01/2012–12/2020) on a 1.5 Tesla scanner (Magnetom Avanto, Siemens Healthcare, Erlangen, Germany) or a 3 Tesla scanner (Magnetom Skyra, Siemens Healthcare, Erlangen, Germany). The image material is archived for analysis in the hospital’s own Picture Archiving and Communication System (PACS) (IMPAX EE R20 XVIII SU1 AGFA, Healthcare N.V.; Mortsel, Belgium 2008–2019). After excluding patients based on the criteria (incomplete examinations, deviating MRI sequences, poor quality images, and evaluation of the epiphysis due to pathology not possible) 848 MRI examinations remained with a total of 431 left knee and 417 right knee acquisitions. Evaluation was conducted in coronal orientation and if coronal images were not available in sagittal orientation.

**Table 1:** Tabular representation of pathologies of patients with MRI images of the knee. Category 1–14 describe the 14 different indications for testing in a total = 900 cases. MRI images of the knees of patients aged 10 to 28 years. MRI: magnetic resonance imaging, n: number

Category	Description of pathology	Number (*n*)
1	Distorsion/Trauma	484
2	Osteomyelitis	23
3	Torsion angle measurement	12
4	Osteochondrosis dissecans	11
5	Synovitis	23
6	Study	37
7	Arthritis	23
8	Benign tumor	54
9	Malignant tumor	46
10	Tendinopathy	13
11	Osteonecrosis, Morbus Osgood-Schlatter disease	48
12	Soft tissue (Neurofibroma, Hoffa-Syndrom, Chondropathia, Baker cyst)	40
13	without pathological findings: gonalgia of unclear etiology, unstable personality disorder	78
14	Meniscal injuries without trauma	8

### MRI classifications

**Fig. 1:**
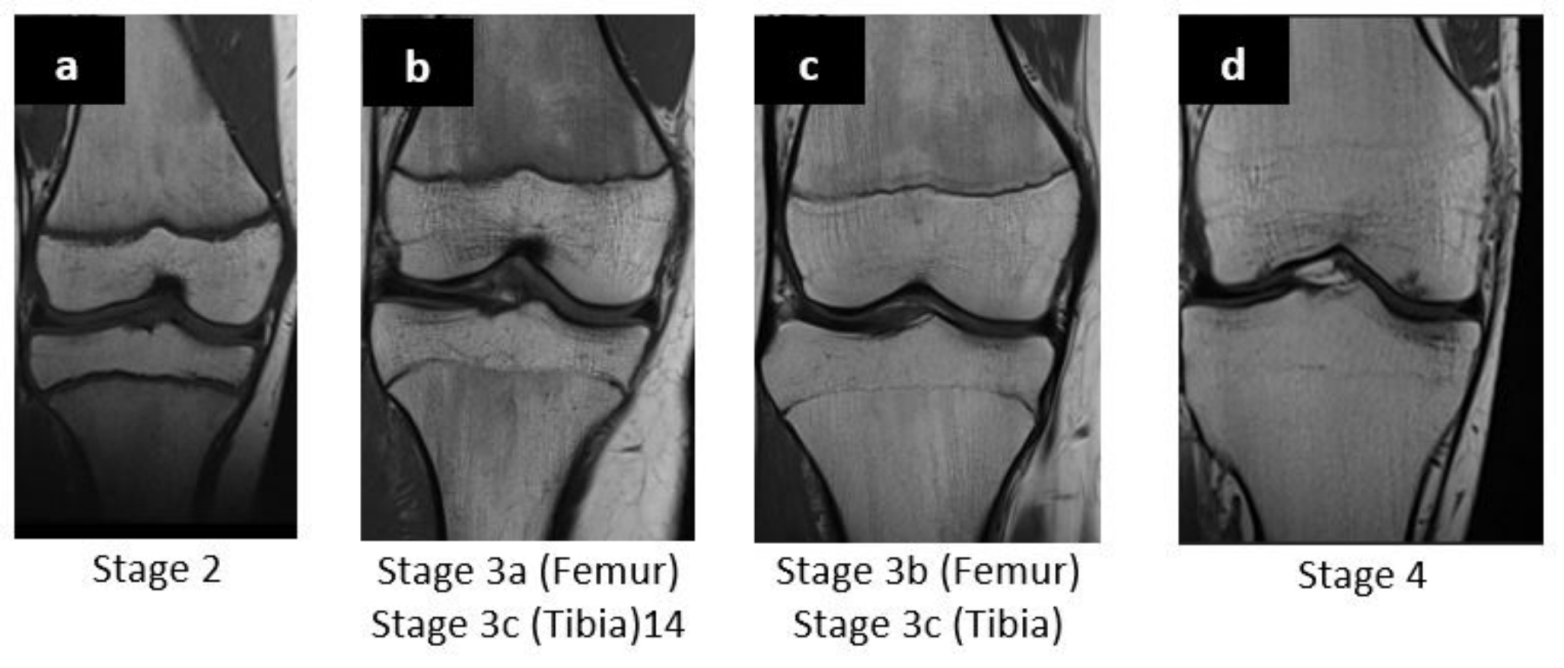
Coronary MRI images in T1-TSE sequence. **a**: Distal femur and proximal tibia of a 10-year-old female patient in stage 2. **b**: Distal femur and proximal tibia of a 14-year-old-female patient. **c**: Distal femur and proximal tibia of a 17-year-old male patient in stage 3b. **d**: Distal femur and proximal tibia of a 21-year-old male patient in stage 4. MRI: Magnetic Resonance Imaging, TSE: Turbo Spin Echo

The MRI datasets were evaluated according to the respective proposed classifications of ossification stages by Ottow et al. (2017) and Vieth et al. (2018) [5, 15]. In a prospective cross-sectional cohort study, Ottow et al. (2017) investigated the relevance of bony fusion of the distal femoral and proximal tibial epiphyses [5]. Based on the well-known classification of Schmeling et al. (2010), the staging is divided into 5 stages based on the ongoing bony fusion of the epiphysis. Stage 2 and 3 are further divided into a, b, and c based on the classification of Kellinghaus et al. (2010). Therefore, Ottow et al. (2017) proposed a MRI classification that includes a total of 11 stages. Figure 1 shows examples of the present studies` cohort with different ossification according to the classification of Ottow et al.

**Fig. 2:**
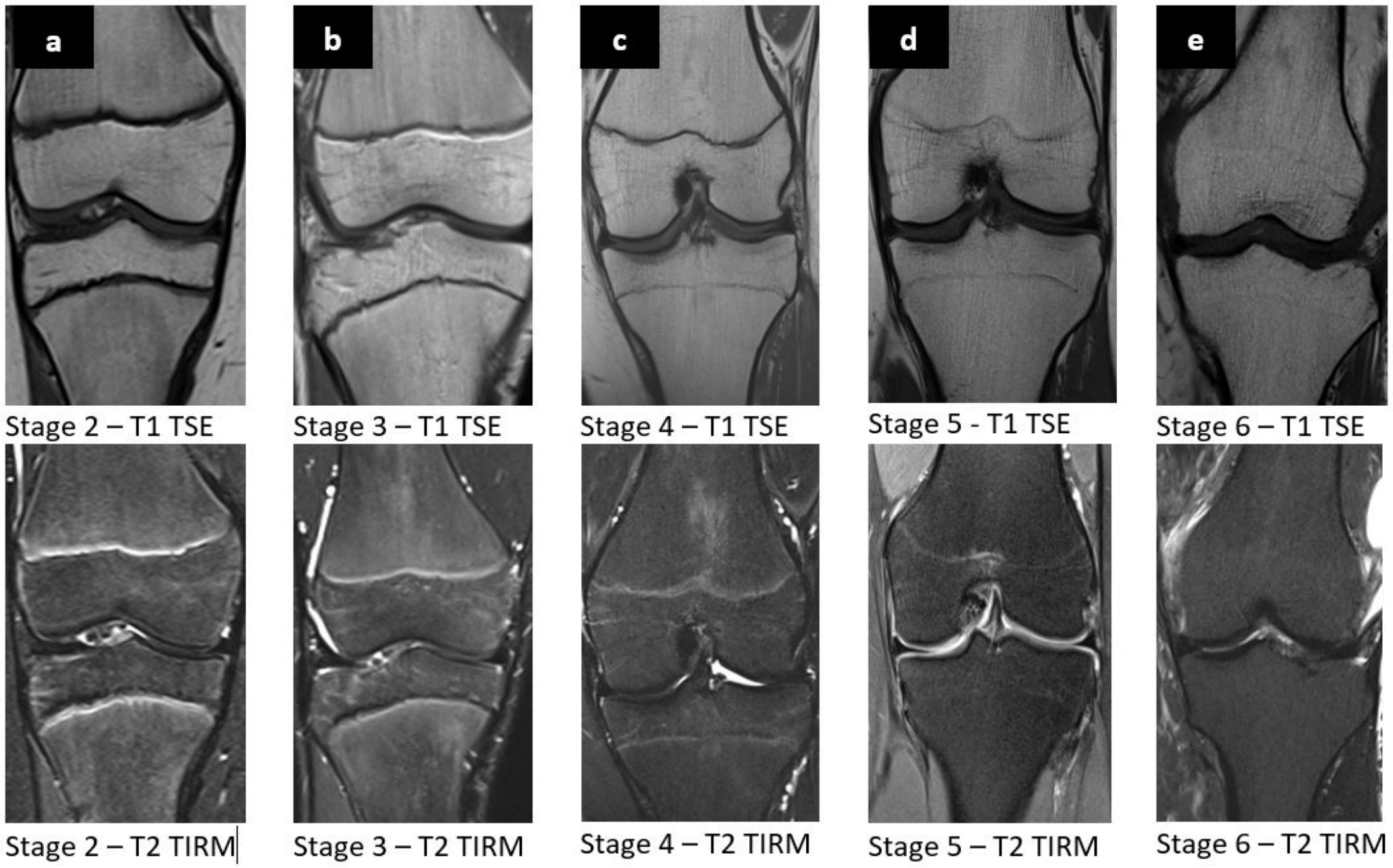
Coronary MRI images in T1-TSE sequence (first row) and T2-TIRM sequence (second row). **a**: distal femur and proximal tibia in in stage 2 of a 10-year-old male patient; **b**: distal femur and proximal tibia of a 14-year-old male patient in stage 3; **c**: distal femur and proximal tibia of a 14-year-old female patient in stage 4; **d**: distal femur and proximal tibia of a 17-year-old female patient in stage 5; **e**: distal femur and proximal tibia of a 24-year-old female patient in stage 6; MRI: Magnetic Resonance Imaging, TSE: Turbo Spin Echo, TIRM: Turbo Inversion Recovery Magnitude

The classification of Vieth et al. uses the T1-weighted and the edema-sensitive T2 spectral presaturation with inversion recovery (SPIR) sequences. The classification is made in 6 stages. Stages 5 and 6 have the same appearance in the T1 sequence and cannot only be differentiated by the T2 sequence. Thereby, a discontinuous linear hyperintense signal is seen in the edema-sensitive T2 in stage 5, which is absent in stage 6. Figure 2 illustrates examples of the different stages from the present studies cohort according to the classification of Vieth et al.

The images underwent initial assessment by a trained examiner with one year of experience in musculoskeletal MRI diagnostics. Subsequently, 114 randomly selected cases (13,4% of all cases) were reevaluated after a six-month interval. A second expert in musculoskeletal radiology (ten years of experience in musculoskeletal MRI diagnostics), unaware of the previous stage determinations, independently reviewed the same set of 114 cases.

### Statistics

Data were analyzed using IBM-SPSS-Statistics-26 (IBM Corp., Armonk, NY). The correlation of results from bone age determination with chronological age was illustrated using box plots. Sex differences were analyzed using the Mann-Whitney U-test. Intra-observer and inter-observer reliability were calculated using Cohen’s kappa coefficient. Interpretation of the calculated Cohens kappa coefficients (κ) was performed according to Landis and Koch [16].

## Results

### Patients´ demographic

Table 2 shows that the sex distribution was balanced at almost every age. Out of 900 patients, 356 patients were under 18 years old and 544 were over 18 years old. Table 3 illustrates a detailed comparison of the number (n) and percentages of both sexes. The distribution of the number between the sexes was similar in each age group, but the total of individuals studied in the age group ≥ 21 years was twice as high as in the other age groups. This fact was due to the age range, which included the age group ≥ 21 years all individuals from 21 years to 28 years.

**Table 2:** Age and sex distribution of patients aged 10 to 28 years with an MRI scan of the knee. Number of total cases = 900 cases. MRI images of the knees of patients aged 10 to 28 years. MRI: magnetic resonance imaging

Chronologic age	*n* (male)	*n* (female)
10	7	16
11	11	21
12	24	20
13	22	16
14	25	27
15	29	28
16	32	22
17	28	28
18	36	18
19	29	23
20	23	23
21	26	28
22	25	22
23	29	18
24	34	16
25	32	31
26	22	19
27	30	15
28 total (∑)	31 ∑ 495	14 ∑405

**Table 3:** Sex distribution of patients aged 10 to 28 years with an MRI scan of the knee, in correlation with defined age groups. Presentation of the number(s) and percentages [%] of a total of 900 cases. MRI images of the knees of patients aged 10 to 28 years. MRI: magnetic resonance imaging

Age group	Sex *n* (male), [percentage]	*n* (female), [percentage]
< 14	64 [12,9%]	73 [18,0%]
14–17	114 [23,0%]	105 [25,9%]
18–20	88 [17,7%]	64 [15,8%]
≥ 21	229 [46,3%]	163 [40,3%]
total (∑)	∑ 495 [100%]	∑ 405 [100%]

### Comparison of ossification stages with chronological age

The identified stages were subdivided by sex according to the respective method and correlated with corresponding chronological age. For clarity, the graphical representation of the data in the following sections has been narrowed down to the distal femur. No statistically significant differences were detected between the right and left knee joints. Therefore, a separate, side-by-side presentation of the results was considered redundant. In 62 patients, both sides were scanned on the same day. Upon direct comparison, the stages according to Ottow et al. and Vieth et al. were found to be identical.

### Classification system I

**Fig. 3:**
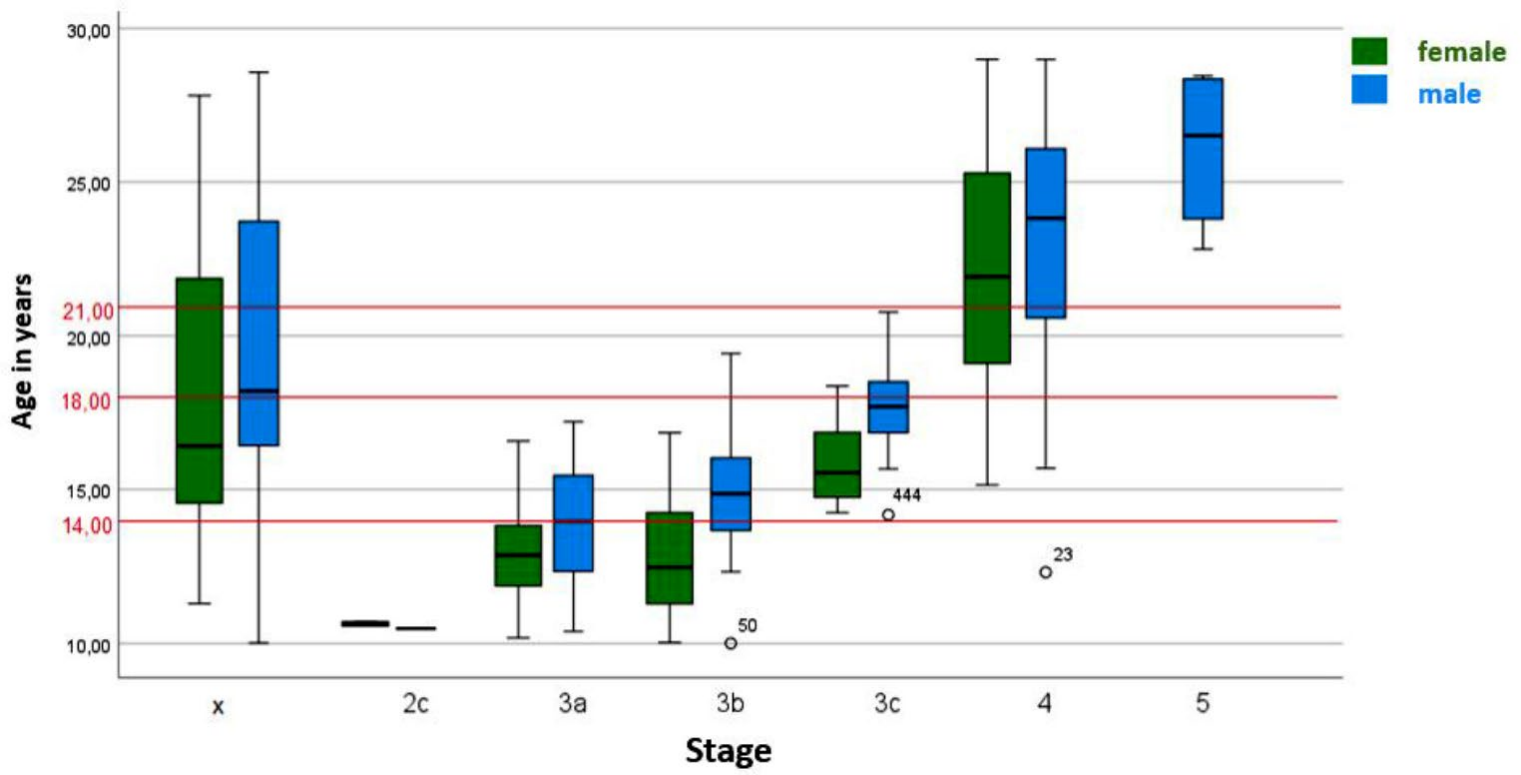
Classification of MRI images of the knees (*n* = 900) into stages 2c to 5 according to the classification system according to Ottow et al. The graph illustrates the distribution of the stages of the distal femur over age. the “x” indicates the excluded examinations. The red lines indicate the forensically relevant age limits. Stage 5 was not assigned to the female patients in any MRI Scan for the distal femur, so the box plot is missing at the corresponding location. The white dots indicate statistically deviating values. MRI images of the knees of patients aged 10 to 28 years. MRI: Magnetic Resonance Imaging, n: number

Figure 3 describes the relationship between the identified stages for the distal femur and chronological age according to the classification of Ottow et al..

For the 495 male patients, stage 2c was assigned once, the patient was 10.49 years old at the time of examination. Stage 3a was assigned 72 times in total, the youngest was 10.39 years old and the oldest was 17.20 years old. Stage 3b could be assigned 55 times, the youngest patient was 10.00 years old and the oldest was 19.43 years old. However, 54 of the patients were younger than 18 years at the time of the study. Only one was 19.43 years old. If this case is bracketed, the highest age for stage 3b was 17.91 years.

Stage 3c was assigned a total of 41 times, the youngest was 14.18 years old and the oldest was 20.77 years old. Stage 4 was assigned 293 times, the youngest was 12.31 years old and the oldest was 26.08 years old. Among the 293 cases that received stage 4, only one patient was under 14 years old. If this case is bracketed, the youngest was 16.07 years old. Stage 5 was assigned four times, the youngest patient was 22.82 years old and the oldest patient was 28.46 years old. The exact distribution of stages is listed in Table 4.

**Table 4:** Classification of MRI images of the knees (*n* = 900) into stages 2c to 5 according to the classification system according to Ottow et al. The graph illustrates the distribution of the stages of the distal femur over the age. The “x” indicates the excluded examinations. The red lines indicate the forensically relevant age limits. Stage 5 was not assigned to the female patients in any MRI scan for the distal femur, so the box plot is missing at the corresponding location. The white dots indicate statistically deviating values. MRI images of the knees of patients aged 10 to 28 years. MRI: magnetic resonance imaging, n: number

		Age in years – male		
		Number of cases (n)	Minimum	Maximum
Proximal Tibia Stages	X	35	10,02	28,58
	2c	2	10,49	11,52
	3a	56	10,00	17,20
	3b	61	11,32	17,91
	3c	42	14,18	20,77
	4	293	12,31	28,99
	5	2	23,14	25,39
Proximal Fibula Stages	X	40	10,02	28,58
	2c	19	10,39	13,98
	3a	71	10,00	17,20
	3b	31	12,36	19,43
	3c	40	14,18	20,59
	4	287	12,31	28,99
	5	7	22,82	28,47
Distal Femur Stages	X	24	10,02	28,58
	2c	1	10,49	10,49
	3a	72	10,39	17,20
	3b	55	10,00	19,43
	3c	41	14,18	20,77
	4	297	12,31	28,99
	5	4	22,82	28,46

Stage 2c was assigned twice in the evaluation of MRI scans of 405 female patients. At the time of examination, the two female patients were 10.57 years old and 10.71 years old. Stage 3a was assigned a total of 48 times, the youngest was 10.18 years and the oldest was 16.58 years. Stage 3b was awarded 45 times, the youngest patient was 10.03 years old and the oldest was 16.85 years old. Stage 3c was awarded 22 times in total, the youngest was 14.25 years old and the oldest was 18.36 years old. Stage 4 was assigned 258 times, the youngest was 15.15 years old and the oldest was 28.99 years old. The exact distribution of the stages is listed in Table 5.

**Table 5:** Tabular representation of the minimum and maximum age and the resulting mean values and standard deviations in 405 female patients whose MRI images of the knees were evaluated using the classification system according to Ottow et al. Numbers of cases (n) describes the distribution of cases per stage. “X” marks the excluded cases. MRI images of the knees of patients aged 10 to 28 years. MRI: magnetic resonance imaging

Age in years = female				
		Number of cases (n)	Minimum	Maximum
Proximal Tibia Stage	X	28	11,29	27,81
	2c	2	10,25	10,71
	3a	51	10,03	15,82
	3b	36	10,23	16,85
	3c	37	13,63	18,36
	4	244	14,50	28,99
	5	1	27,49	27,49
Proximal Fibula Stage	X	21	11,29	27,81
	2c	18	10,18	14,43
	3a	55	10,03	16,85
	3b	25	10,23	18,36
	3c	29	14,25	19,97
	4	235	15,26	28,99
	5	10	24,25	28,48
Distal Femur Stage	X	28	11,29	27,81
	2c	2	10,57	10,71
	3a	48	10,18	16,58
	3b	45	10,03	16,85
	3c	21	14,25	18,36
	4	260	15,15	28,99

**Fig. 4:**
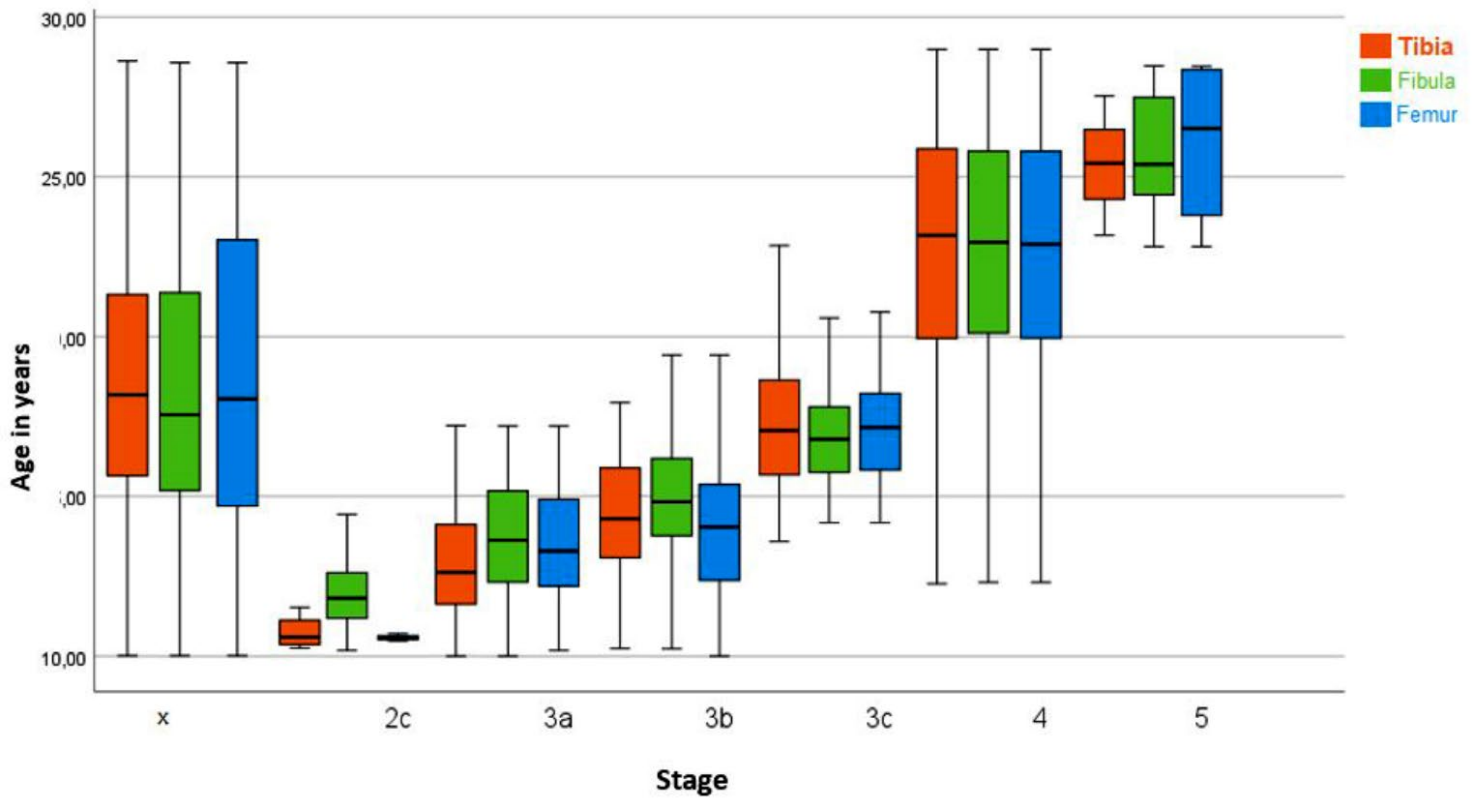
Classification of MRI images of the knees (*n* = 900) into stages 2c to 5 according to the classification system according to Ottow et al. The graph illustrates the age distribution for each stage and long bone. The “X” indicates the excluded examinations. MRI images of the knees of patients aged 10 to 28 years. MRI: Magnetic Resonance Imaging, n: number

Figure 4 illustrates the results of the three epiphyses (distal femur, proximal tibia, and proximal fibula) considering the chronological age at the different anatomical locations of the different stages. All differences are not significant. However, differences could be obtained in stages 2c, 3a, 3b and smaller in stages 3c and 5. In stage 4, only minimal differences were found between the epiphyses.

### Classification system II

**Fig. 5:**
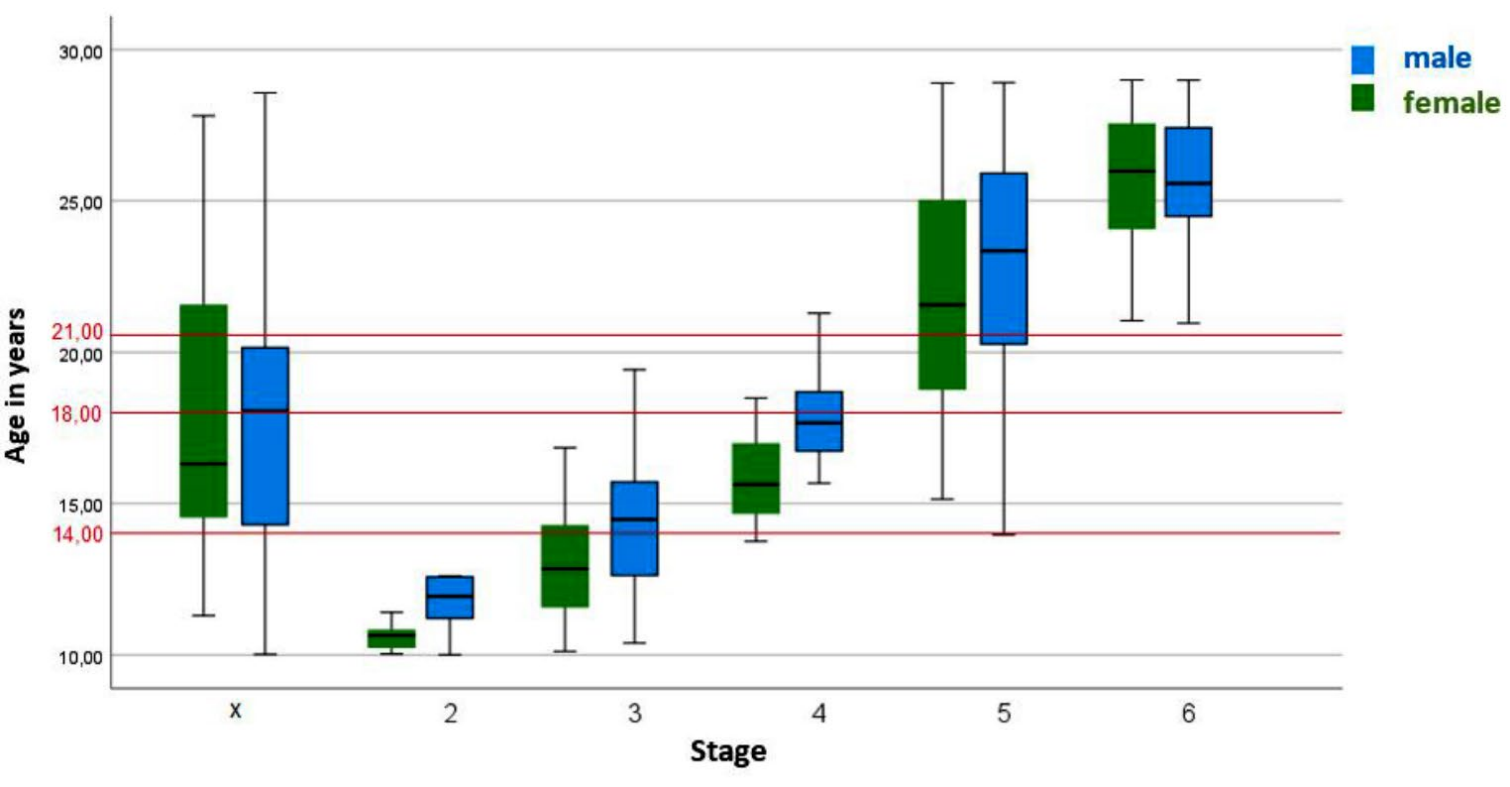
Classification of MRI images of the knees (n = 900) into stages 2 to 6 according to the classification system according to Vieth et al. The graph illustrates the distribution of the stages of the distal femur over age. The “x” indicates the excluded examinations. The red lines indicate the forensically relevant age limits. MRI images of the knees of patients aged 10 to 28 years. MRI: Magnetic Resonance Imaging, n: number

Figure 5 describes the relationship between the identified stages for the distal femur and chronological age according to the classification of Vieth et al.

The youngest male in stage 2 was 10.00 years old and the oldest was 12.61 years old. Stage 3 was assigned in a total of 119 MRI scans, the youngest patient was 10.39 years old and the oldest was 19.43 years old. Stage 4 could be established 47 times in the examinations, the youngest was 15.67 years old and the oldest was 21.28 years old. Of the 47 examinations that counted as stage 4, 45 examinations were under 21 years old, only two were older than 21 years, the exact ages were 21.08 years and 21.28 years. Stage 5 was determined a total of 254 times, the youngest in this stage was 13.96 years old and the oldest was 28.91 years old. Stage 6 was determined a total of 45 times, the youngest was 20.96 years old and the oldest was 28.99 years old. The exact distribution of the stages is listed in Table 6.

**Table 6:** Tabular representation of the minimum and maximum age and the resulting mean and standard deviations of 495 male patients whose MRI images of the knees were evaluated using the classification system according to Vieth et al. Number(s) describes the distribution of cases per stage. “X” marks the excluded cases. MRI images of the knees of patients aged 10 to 28 years. MRI: magnetic resonance imaging

		Age in years – male		
		Number of cases (n)	Minimum	Maximum
Proximal Tibia Stages	X	35	10,02	28,58
	2	6	10,00	14,64
	3	108	10,39	17,91
	4	47	15,15	21,28
	5	212	13,96	28,91
	6	84	18,34	28,99
Proximal Fibula Stages	X	40	10,02	28,58
	2	37	10,00	14,64
	3	75	11,33	18,27
	4	46	15,67	21,28
	5	190	13,96	28,91
	6	100	17,02	28,99
Distal Femur Stages	X	24	10,02	28,58
	2	5	10,00	12,61
	3	119	10,39	19,43
	4	49	15,67	21,28
	5	253	13,96	28,91
	6	46	21,04	28,99

When MRI scans of 405 female patients were evaluated, stage 2 was assigned a total of 6 times, the youngest in this stage was 10.03 years old and the oldest was 11.41 years old. Stage 3 was assigned a total of 91 times; the youngest was 10.12 years old and the oldest was 16.85 years. Stage 4 could be assigned 24 times, the youngest was 13.75 years old and the oldest was 18.49 years old. Of the 24 examinations assigned stage 4, 22 were under 18 years old, two of them were older than 18 years, and the exact ages were 18.36 years and 18.49 years. Stage 5 was assigned a total of 223 times, the youngest was 15.15 years old and the oldest was 28.90 years old. Stage 6 was assigned 31 times, the youngest was 21.05 years old and the oldest was 28.99 years old. The exact distribution of the stages is listed in Table 7.

**Table 7:** Tabular representation of the minimum and maximum age and the resulting mean and standard deviations of 405 female patients whose MRI images of the knees were evaluated using the classification system according to Vieth et al. Number(s) describes the distribution of cases per stage. “X” marks the excluded cases. MRI scans of the knees of patients aged 10 to 28 years. MRI = magnetic resonance imaging

		Age in years – female		
		Number of cases (n)	Minimum	Maximum
Proximal Tibia Stages	X	28	11,29	27,81
	2	5	10,25	11,41
	3	78	10,03	16,85
	4	41	13,59	21,97
	5	188	14,50	28,99
	6	59	19,72	28,69
Proximal Fibula Stages	X	21	11,29	28,69
	2	48	10,03	14,43
	3	49	10,83	18,36
	4	29	13,75	21,56
	5	165	15,26	28,90
	6	74	17,76	28,99
Distal Femur Stages	X	28	11,29	27,81
	2	7	10,03	11,41
	3	91	10,12	16,85
	4	24	13,75	18,49
	5	223	15,15	28,90
	6	31	21,05	28,99

**Fig. 6:**
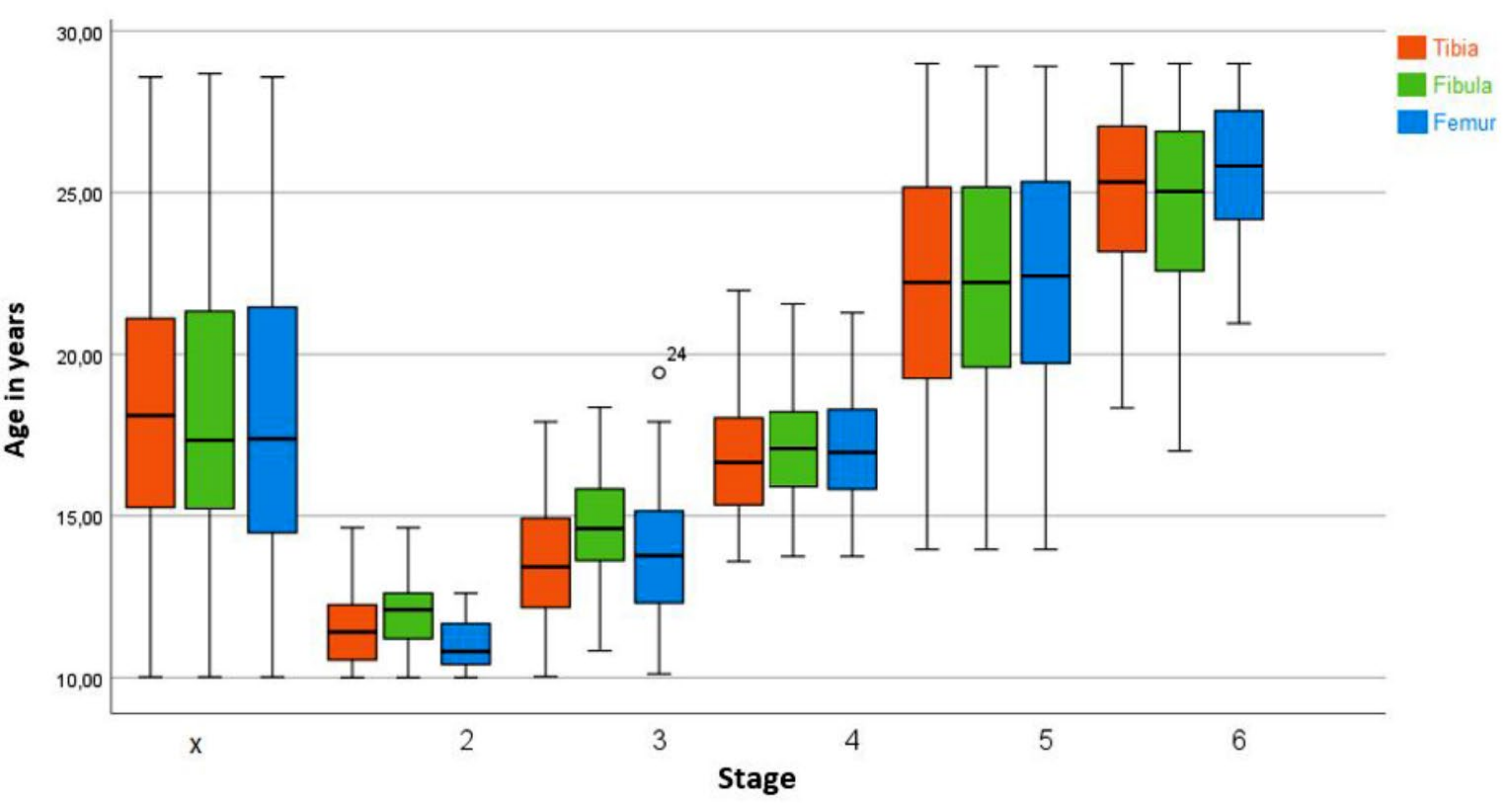
Classification of MRI images of the knees (*n* = 900) into stages 2 to 6 according to the classification system according to Vieth et al. The graph illustrates the age distribution for each stage and long bone. The “X” indicates the excluded examinations. The white dots indicate statistically deviating values. MRI images of the knees of patients aged 10 to 28 years. MRI: Magnetic Resonance Imaging, n: number

The results of the three epiphyses (distal femur, proximal tibia, and proximal fibula) are shown in Fig. 6. Considering the chronological age at the different anatomical locations of the different stages no significant differences could be seen. However, there are differences in stages 2 and 3, and smaller differences in stages 4 and 6. In stage 5, only minimal differences were found between the epiphyses.

### Intra- and Inter-observer agreement 

We found a very good intra-observer agreement after calculating Cohen´s kappa coefficient for the distal femur (κ = 0.953), the proximal tibia (κ = 0.906) and the proximal fibula (κ = 0.884) for the classification of Ottow et al. The results for the classification of Vieth et al. were comparably good concerning the distal femur (κ = 0.941), the proximal tibia (κ = 0.919) and the proximal fibula (κ = 0.897).

After calculating Cohen´s kappa we found a very good inter-observer agreement concerning the distal femur (κ = 0.858), the proximal tibia (κ = 0.816) and the proximal fibula (κ = 0.860) for the classification of Ottow et al. and concerning the distal femur (κ = 0.877), the proximal tibia (κ = 0.879) and the proximal fibula (κ = 0.880) for the classification of Vieth et al.

## Discussion

### Use of MRI to determine age

Many studies have repeatedly demonstrated that ossification of the epiphysis is a process from which information can be used to estimate age [17]. For instance, Fan et al. showed that chronological age correlates well with ossification of the epiphyses in the knee region [18]. However, many earlier studies relied on X-ray images analyzed according to the standards of Greulich and Pyle or Tanner and Whitehouse [1, 19, 20].

X-ray images of the hand, orthopantomography of the jaw and CT examinations of the clavicle epiphyses are included in the AGFAD recommendations. These examinations for forensic age assessment are not medically indicated. The use of ionizing radiation for forensic age determination is always the subject of controversy. Even if the radiation exposure is low, especially for hand X-rays, it is desirable to avoid exposure. Alternatively, radiation-free imaging procedures analogous to the recommendations of the AGFAD, such as sonography of the wrist and MRI of the clavicle, are available [19, 21, 22]. The use of MRI of other body regions, such as the knee joint for forensic age diagnosis, is also the subject of numerous studies [23].

However, in addition to the advantage that ionizing radiation can be avoided, other factors must also be considered. These include longer scanning times, relative contraindications, higher costs and lower availability. In addition, patient age, compliance and any phobic reactions, which can lead to artifacts and limited assessability, must also be taken into account [24].

Methods focusing on the evaluation of MRI images of the knee are based on the evaluation standards of Kellinghaus and Schmeling [6, 25]. The ossification process of the knee and the good correlation with chronological age could be demonstrated by analyzing MRI images as well as X-ray images. In addition, MRI, being a cross-sectional imaging technique, provides multiple two-dimensional images of body areas, offering more comprehensive information compared to conventional X-ray projection images. This makes it possible to recognize incomplete or early-stage epiphyseal fusion on at least one image within the series. Conventional X-ray images offer only a singular perspective for analysis (24; 25). Therefore, age estimation using MRI scans should be considered as an alternative to X-ray images. As Fan et al. correctly stated, the knee joint offers the possibility to examine three epiphyses of long bones (distal femur, proximal tibia and proximal fibula) in one image [18]. All three long bones were assessed in the current study. While Ottow et al. expressed concerns about the reliability of assessing the proximal fibula epiphysis due to a limited series of images where it appears [5], our results demonstrate that the ossification of the fibular epiphysis can indeed be reliably evaluated. However, the results of the proximal fibular epiphysis are different to the results of the proximal tibial and distal femoral epiphysis.

### Duration of the examination and evaluation

It takes a few minutes to produce an MRI image. In our study the pure acquisition time for the T1 TSE was 2 min 54 s (3 Tesla), 3 min 1 s (1,5 Tesla) and the T2 TIRM 2 min 26 s, respectively. According to the literature, a pure acquisition time of 2 min and 45 s applies for the T1-VIBE sequence (Volumetric Interpolated Breathhold Examination) for age estimation of the hand. In addition, there is an undefined period of time for the correct preparation of the patient [10]. In this context, it should also be mentioned that image acquisition in general has become faster, partly due to the use of artificial intelligence [26]. For instance, a study by Neumayer et al. demonstrated a significant reduction in acquisition time for MRI-based forensic age estimation of the hand through data undersampling [27]. It is important to note that the knee joint is an easily accessible region, which is less susceptible to movement artefacts and often offers no variations, unlike the medial epiphyses of the clavicles [24]. The evaluation according to Ottow et al. required an average time of 8.8 s. The evaluation according to Vieth et al. required an average time span of 10.6 s, this longer time span is due to the observation of T1 and additionally T2 sequences at stage 5 and stage 6.

### Results from Vieth et al.

The results of this study indicate that the proposed method by Vieth et al. is effective in accurately determining age limits. Specifically, age limits 14 and 21 were successfully established for both sexes. The age limit 18 could be determined for the female patients with stages 2–4. If stage 6 was classified at the distal femur in both sexes, all patients were older than 18 and also older than 21 years, even if the minimum age of stage 6 was very close to the age limit of 21 (female 21.05 and male 21.04).

The statistical bias described by Vieth et al. at the age limits of 14 and 21 years was not observed in this study, as the upper age limit for this study was 28 years instead of 24 years, as in Vieth et al. Furthermore, the present study has a sufficient number (*N* = 77) of cases in stage 6. In addition, the lower cut-off for the entire cohort in this study was also set at 10 years instead of the lower cut-off of 12 years selected by Vieth et al. Thereby, the selection bias for stages 2 and 3 is reduced. The proposal of Vieth et al. to consider the minimum age for stages 2 and 3 as well as the maximum age for stage 6 as artificial limits could be confirmed, as the limits adjusted with the extension of the age limits.

The results of Vieth et al., which were presented using the “minimum age concept”, were confirmed in the present study. For the distal femur, it was found that stage 6 was assigned in all cases to persons over 18 years of age in both sexes, as the youngest age was 21.05 years for females and 21.04 years for males. Stage 4 could always be assigned to males over 14 years of age, as the youngest age was 15.67 years. Stage 5 could always be assigned to females over 14 years of age, as the youngest age was 15.15 years. When considering the proximal tibia and fibula, stage 5 was assigned to females over 14 years of age in all cases and stage 4 was assigned to males over 14 years of age in all cases. Stage 6 could only be assigned for the proximal tibia in all cases to persons over 18 years of age for both sexes.

To summarise, 100% of stage 2 patients were under 14 years of age in both sexes and 100% of stage 3 patients were under 21 years of age in both sexes. Stage 4 could always be assigned to males over 14 years of age and stage 5 could always be assigned to females over 14 years of age. With stage 6, the completion of the age of 21 could be determined 100% of the time in both sexes. These results apply when the distal femur is assessed. When considering the proximal tibia and fibula, stage 5 was assigned to females over 14 years of age in all cases and stage 4 was assigned to males over 14 years of age in all cases. In both sexes, all individuals in stage 6 were older than 18 years only at the proximal tibia. For males, the minimum age of 18.34 years in stage 6 was also very close to the age limit of 18 years. This once again illustrates the importance of exceeding or falling below certain stages with regard to forensic age determination. Furthermore, it was observed that for each stage of the three long bones reached, the mean age was younger in females than in males. This finding has also been documented in other studies [3, 5, 18, 28–31].

It is important to note that if a stage cannot be assigned, the chronological age of the person does not automatically fall beyond the corresponding age limit. For example, a person with stage 2 is 100% under 14 years old, but it should not be assumed that if stage 2 is not assigned, the person is over 14 years old.

### Results from Ottow et al.

Ottow et al. found that significant differences between women and men in the ossification process of the distal femur are only found from the age of 13. Female patients reached stage 3b at 14.73 years, while male patients only reached stage 3a at 17.77 years [31, 32]. Similar results can be reported in the present study. According to Ottow et al., female patients reached stage 2c at 12.11 years, in the present study at 10.57 years (mean average age of the patients was 10.64 years). Stage 3a was reached at 13.39 years (Ottow et al.) and in the present study at 10.18 years (mean average age 12.91 years). The variations in minimum age across the different stages can be explained by the diverse age groups considered, leading to potential biases in the results, especially close to the age limits. From stage 3b onwards, the results of Ottow et al. were confirmed in that a significant difference between the sexes could be recognized after the age of 13.

According to Ottow et al., the age of 14 years in males can be determined via stage 3c of the distal femoral epiphysis and the proximal tibial epiphysis. Similar results were obtained by Krämer et al. and partly by Jopp et al. [5, 30, 33]. Krämer et al. showed that the completion of the 14th year of life could be determined with stage 4 in females and with stage 3c in males [30]. The results of the present study are consistent with the data from the studies described above, as the age of 14 years could be determined for the distal femur in males with stage 3c. The youngest age in this case was 14.18 years. The same applies to the proximal tibia and fibula, in both cases, the youngest age of males with stage 3c was 14.18 years. However, in contrast to the results of other studies, in the present study the completion of the 14th year of life could also be determined for the distal femur in females with stage 3c, as the youngest age was 14.25 years. Based on the assessment of the proximal tibia and fibula, it is possible to identify females over 14 years with stage 4. Persons under 14 years could be identified by stage 2c in all three long bones. The exact age limits of all stages and long bones are shown in Fig. 6.

Ottow et al. also described that it was not possible to reliably determine the age of 18 years in both sexes due to the defined stages and the associated sub-stages, as the earliest documented stage 4 of the distal femoral epiphysis in males was under 18 years in several cases. The results of the present study contradict the finding described above. Among 356 cases who were under 18 years of age at the time of the study, 222 cases were categorized as stage 2c, 3a or 3b. Subjects with stage 3c were always under 21 years old. Stage 4 was assigned to patients over 18 years of age in 89.2% of cases and stage 5 was assigned to patients over 21 years of age in 100% of cases. Ottow et al. assumed that the minimum age for stage 5 was over 24 years for both sexes. Due to their chosen age range of 12 years to 24 years, a bias in the results close to the age limits was assumed. These observations and assumptions are consistent with those of Krämer et al. [19, 30, 31]. In the present study, due to the extension of the maximum cut-off value to 28 years, it could be shown that stage 5 is above the age of 21 years in both sexes. Stage 1-2b could not be assigned despite the minimum threshold of 10 years.

In summary, upon closer examination, not all the results from Ottow et al. could be confirmed in our study. However, it was possible to determine that if only the distal femur was considered, 100% of under 14-year-olds could be identified for both sexes using stage 2c and 100% of under 18-year-olds for both sexes using stage 3a. Stage 3b could be assigned 100% to females under the age of 18. Stage 3c was assigned 100% to over 14-year-olds on the one hand and always to under 21-year-olds in both sexes on the other. Stage 5 could be assigned to males over the age of 21. Accordingly, the classification by Ottow et al. was able to determine the completion of the 18th and 21st year of life in both sexes based on the distal femur.

When looking at the proximal tibia and fibula, sex-specific differences were recognizable, while stage 3c could always be assigned to males over 14 years of age, stage 4 was assigned to females over 14 years of age. Stage 5 could be assigned to persons over 21 years of age in both sexes. Furthermore, it was observed in other studies that for each stage of the three tubular bones reached, the mean age was lower in females than in males. As described by Ottow et al., this corresponds to the results of several studies [3, 5, 18, 28–30]. It should also be noted at this point that if, for example, stage 3a is assigned, the person is 100% under 18 years of age, but it cannot be stated that at higher stages the person is automatically over 18 years of age. The definition of the age groups must therefore be considered when interpreting the different stages.

One would assume similar minimum ages in both classifications for the respective stages considering that stage 4 (Ottow et al.) and stage 5 (Vieth et al.) are basically the same in the T1w-sequence, when ignoring the water sensitive sequence. In males, the minimum age in stage 4 (Ottow et al.) was lower with 12.31 years for all three bones than in stage 5 (Vieth et al.) with 13.96 years. This difference could possibly be due to the additional assessment of the TIRM-sequence for the classification according to Vieth et al. In females, the minimum age was the same for all three bones and the maximum age differed only minimally by 0.09 years.

The two referenced classifications were initially applied to coronal MR images. In our study, both coronal and sagittal orientations were included in the analysis. This could be a possible reason for the deviations in the minimum ages, even though the classifications primarily refer to the assessment of the epiphysis and were further developed from the classification of the ossification of the epiphysis of the clavicle.

In the “classic” method of bone age estimation following the Greulich and Pyle (GP) standards using hand radiographs, the non-dominant left side is typically utilized. This approach estimates bone age by comparing the radiographs of the subject’s non-dominant wrist with the nearest matching reference radiographs provided in the atlas. The rationale behind this choice lies in the fact that the majority of individuals are right-dominant, making the left hand less susceptible to traumatic changes or deformities. While some studies have reported differences in bone age between the right and left hand in direct comparisons, others have found no such distinctions [34–38]. In our current study, even among the 62 patients who underwent MRI scans of both knee joints on the same day, the stage was consistently identical for both sides, as per the criteria outlined by Ottow et al. and Vieth et al.

The intra-observer variability results, although slightly lower compared to Ottow et al. and Vieth et al., still demonstrate a high level of accuracy, rating as very good. Notably, despite differences in experience between the two investigators, the intra-observer variability results remained consistently very good. These findings suggest that both classifications yield reliable results even with varying levels of experience. In a study concerning bone age determination utilizing the Greulich/Pyle method, Lynnerup et al. demonstrated that examiner experience did not significantly impact evaluation accuracy in a direct comparison [39]. However, whether this principle extends to MR classifications, as employed in our study, warrants further investigation through additional comparative studies.

In our study, we employed MR sequences as outlined in the original studies by Ottow et al. and Vieth et al., utilizing T1 turbo spin-echo (TSE) and T2 TSE with fat suppression via spectral pre-saturation with inversion recovery (SPIR). T2-weighted images proved crucial, particularly in distinguishing between stage 5 and stage 6 according to the Vieth classification. Meanwhile, T1-weighted images offered exceptional clarity in analyzing ossified parts of the epiphysis. The utilization of fat-suppressed MR images generally enhance sensitivity for detecting subchondral bone marrow lesions. Furthermore, for bone age estimation, these images facilitate the visualization of fluid signals at the epiphyseal scar, aiding in the differentiation of stages 5 and 6 as per Vieth et al. Beside insensitivity to magnetic field inhomogenity it is important to acknowledge extended imaging durationand lower signal-to-noise ratio compared to alternative fat suppression techniques [40].

Instead of utilizing the fat-suppressed T2-weighted images recommended by Vieth et al., Has et al. opted for proton-weighted sequences (PD) without direct comparison to the fat suppression technique via SPIR as proposed in the original publication [41]. While articular cartilage differs significantly, the vascularization of epiphyseal cartilage offers an opportunity for imaging during human skeletal development. In an ex vivo study, Ellermann et al. successfully visualized vascularized epiphyseal cartilage using gradient-recalled-echo sequences (GRE), allowing for a detailed differentiation between ossified and cartilaginous segments of the epiphysis [42]. This enhanced visualization of the cartilaginous parts could significantly improve differentiation of various stages, particularly in stages 3 a-c by Ottow et al.

A recent review on MRI analysis of the knee for bone age determination underscored the heterogeneity of study populations, grading systems, and MR protocols, leading to limited comparability [43]. Future studies should aim to directly compare the advantages and disadvantages of different MR sequences in age determination to establish recommended MR protocols. This comparative approach would offer valuable insights into optimizing imaging techniques for more accurate bone age assessment.

### Limitations

The impact of actual illnesses or malnutrition in adolescents and young adults on epiphyseal growth requires careful evaluation. In the study at hand, all known potentially influencing factors were documented in the categorization, but not considered in detail. Table 1 lists all indications for the MRI scans. The present study is subject to certain limitations, notably the absence of recorded systemic diseases and medication histories of the included patients. Such factors could potentially impact the ossification process of the epiphysis. Among these variables are growth disorders, hormone therapy, steroid usage, and chemotherapy, all of which may exert significant influence on the epiphyseal ossification.

Despite not taking the individual diseases into account, a reliable categorization of the stages was possible. In the case of pathologies in the used MRIs that impaired the assessment of the epiphyseal joints, the examination was excluded.

The classifications utilized in this study were originally based on MRIs of healthy German volunteers and subsequently applied to a cohort of German patients. Notably, alternative classification systems, like that proposed by Dedouit et al., were not incorporated. Future investigations should delve into comparing these classification methodologies and extend the analysis to encompass diverse ethnic backgrounds [28, 43]. However, the present study shows that the application of classification systems developed on healthy persons can be applied well to German patients with different indications for MRI scans, as the results were largely confirmed.

Another crucial consideration is the assumed correlation between ethnicity and socioeconomic factors influencing bone growth [18, 28, 44]. However, there is scientific disagreement regarding which of the two factors influence bone growth. Since the actual patients in the studies have a high developmental status, the ossification process is completed earlier than patients with lower socioeconomic status. Consequently, an underestimation of age may not be disadvantageous from a forensic point of view [25, 44, 45]. Moreover, defining an individual’s socioeconomic status poses challenges, and even with a successful basic definition, clear assessment and proof can be challenging, especially in the case of refugees.

## Conclusion

The primary objective of this study was to find out to what extent magnetic resonance imaging (MRI) can be used in forensic age diagnostics. Based on this, it was to be investigated whether MRI images of the knee region can be used to determine the forensically relevant age limits to offer a radiation-free examination alternative in forensics in the future.

The present results of a German patient population demonstrate that the determination of forensically relevant age limits is possible. With the classification of Vieth et al. it was possible to ascertain the completion of the 14th and 21st year of life for both sexes and the 18th year of life for the female patients. In addition, with stage 6 at the distal femur all patients in both sexes were older than 18 and also older than 21 years.With the classification of Ottow et al. it was possible to determine completion of the 18th and 21st year of life for both sexes.

The assessment of ossification of the epiphysis in the knee joint using MRI can help to determine legally relevant age limits. The combined application of both examination methods proves to be effective in drawing direct conclusions about chronological age and forensically relevant age limits through the radiation-free assessment of skeletal maturity. The investigated method could therefore be used to support forensic age diagnostics.
