# Correction: Forensic age estimation by MRI of the knee – comparison of two classifications for ossification stages in a German population

**DOI:** 10.1007/s00414-026-03757-6

**Published:** 2026-03-10

**Authors:** V Malokaj, MF Wernsing, SN Kunz, M Beer, D Vogele

**Affiliations:** 1 Department of Diagnostic and Interventional, Radiology University Hospital Ulm, Albert-Einstein-Allee 23, 89081 Ulm, Germany; 2 Institute of Forensic Medicine, Ulm University, Ulm, Germany

**Correction: International Journal of Legal Medicine (2024) 138:2387–2400**

10.1007/s00414-024-03281-5

In the original version of the manuscript, the corresponding author’s given name and family name were swapped. The correct author name should be Daniel Vogele, not Vogele Daniel.

Additionally, the given and family names of the two co-authors have also been swapped: Wernsing MF should be MF Wernsing, and Kunz SN should be SN Kunz.

The original article has been corrected.

